# The Gut–Kidney–Metabolic Axis: Impact of Gut-Derived Uremic Toxins on Insulin Resistance in Diabetic Kidney Disease

**DOI:** 10.3390/ijms27083472

**Published:** 2026-04-13

**Authors:** Charlotte Delrue, Margaux Vinckier, Reinhart Speeckaert, Stefania Marzocco, Marijn M. Speeckaert

**Affiliations:** 1 Department of Nephrology, Ghent University Hospital, 9000 Ghent, Belgium; 2 Department of Dermatology, Ghent University Hospital, 9000 Ghent, Belgium; 3 Department of Pharmacy, University of Salerno, Via Giovanni Paolo II 132, 84084 Fisciano, Italy; 4 Research Foundation-Flanders (FWO), 1000 Brussels, Belgium

**Keywords:** gut–kidney axis, diabetic kidney disease, chronic kidney disease, insulin resistance, uremic toxins, indoxyl sulfate, p-cresyl sulfate, trimethylamine-N-oxide, oxidative stress, microbiota modulation

## Abstract

Chronic kidney disease (CKD), especially diabetic kidney disease (DKD), is characterized not only by progressive loss of renal function but also by profound metabolic disturbances, including insulin resistance (IR). Emerging evidence implicates gut-derived uremic toxins as mediators linking intestinal dysbiosis to metabolic and renal injury. Several microbial metabolites, for example, indoxyl sulfate, p-cresyl sulfate, and trimethylamine-N-oxide, are known to accumulate in CKD due to decreased renal excretion and altered tubular secretion. In vitro and in vivo experiments indicate that these gut-derived nephrotoxins impair insulin signaling pathways in cells. This results in increased production of reactive oxygen species, activation of stress kinases, higher levels of inflammatory cytokines, and inhibitory serine phosphorylation of insulin receptor substrates. Consequently, phosphatidylinositol 3-kinase (PI3K)/protein kinase B (Akt) signaling is impaired, reducing cellular glucose uptake. At the same time, these toxins induce endothelial dysfunction and mitochondrial damage, not only causing systemic IR but also contributing to the progression of kidney disease. Observational data link higher plasma toxin levels with components of IR, rapid loss of renal function as measured by estimated glomerular filtration rate, and a high risk of cardiovascular events in CKD patients. Although causality in humans remains unproven, interventions targeting the microbiota, toxin binding, and oxidative stress pathways show promise. We propose an integrated gut–kidney–metabolic framework in which dysbiosis-driven toxin production may amplify IR and DKD progression.

## 1. Introduction

Chronic kidney disease (CKD) affects 8–16% of the global population and is a major contributor to morbidity and mortality [1]. CKD and diabetic kidney disease (DKD) have become major global problems resulting in late-stage kidney failure and the need for renal replacement therapy, accounting for 30–40% of all patients requiring such treatment [2]. These diseases have a hallmark feature: the kidney’s inability to excrete bioactive solutes, called uremic toxins, which steadily accumulate because the kidneys cannot perform their filtration and excretory functions properly [3]. Uremic toxins arise from endogenous metabolism, dietary intake, and gut microbial activity and include protein-bound solutes such as indoxyl sulfate (IS) and p-cresyl sulfate (PCS), as well as small water-soluble molecules like trimethylamine-N-oxide (TMAO), which gradually accumulate as renal clearance decreases. These substances (uremic toxins) have been associated with the promotion of inflammation, the generation of oxidative stress (via reactive oxygen species [ROS]), and the impairment of vascular function (via endothelial cell activation and/or apoptosis) [1].

The gut–kidney axis has gained increasing attention. It is not only that dysbiosis (i.e., an overabundance of opportunistic bacteria) occurs in CKD, but also that it can significantly accelerate CKD progression. The intestinal epithelial layer can be disrupted when uremia occurs and when epithelial junction proteins are downregulated, creating a “leaky gut” and allowing the entry of microbial by-products and endotoxins into the systemic circulation. Chronic dysbiosis shifts microbial metabolism from predominantly saccharolytic to proteolytic pathways. As a result, uremic toxins will increase, and the amount of beneficial short-chain fatty acids (SCFAs) produced by gut bacteria will decrease [4,5,6].

Parallel to renal and microbiota alterations, insulin resistance (IR) is highly prevalent in CKD and especially in DKD patients, independent of adiposity or classic metabolic syndrome features [7]. Direct metabolic assessment in CKD patients has confirmed reduced insulin sensitivity using clamp-based and surrogate methodologies, supporting the concept that renal dysfunction per se contributes to systemic IR [8]. The main factors contributing to IR in CKD are multiple. They include chronic inflammation and oxidative stress, as well as direct interference with the insulin signaling system [9]. Studies have shown that protein-bound uremic toxins can disrupt intracellular metabolic pathways by increasing ROS levels, causing inflammation, and inhibiting glucose uptake following insulin stimulation [10]. Additionally, recent research indicates that increased levels of uremic toxins in patients with decreased glomerular filtration and altered glucose metabolism are associated with the development of IR [11].

Even though earlier studies have discussed the individual components of the gut-to-kidney connection, uremic toxins from the gut, and IR [1], no study has examined how to integrate these components into a cohesive synthesis of their roles in IR and DKD. This is especially important because DKD is a unique confluence of metabolic and kidney damage, where uremic toxins could accelerate the effects of IR and kidney damage [2]. Most prior studies have focused on renal or cardiovascular outcomes associated with uremic toxins, but have not yet systematically examined how uremic toxins can alter insulin receptor signaling in DKD. We propose a mechanistic framework in which gut-derived uremic toxins may influence metabolic regulation and contribute to additional IR by modulating oxidative, inflammatory, and stress kinase pathways. By explicitly integrating molecular signaling data with clinical IR indices and renal outcomes, this review aims to move beyond descriptive association towards a unified pathophysiological model. Moreover, we suggest that the role of toxins in IR should be viewed not merely as a byproduct of kidney dysfunction, but rather as a potential contributory mechanism that may accelerate progression through the different stages of DKD. In this framework, gut-derived toxins are not merely markers of renal dysfunction but potential modulators of metabolic pathways.

In this narrative review, we aim to (i) summarize current understanding of gut dysbiosis and production of uremic toxins in CKD and DKD, (ii) elucidate mechanistic pathways linking uremic toxins to IR, and (iii) explore emerging therapeutic strategies targeting microbiota, metabolite modulation, and insulin sensitization. By integrating insights from microbiome research, metabolic studies, and renal pathophysiology, we highlight a comprehensive, mechanistically informed model of how gut-derived toxins may bridge metabolic and renal disease processes in DKD. The novelty of this work lies in the explicit integration of intracellular insulin signaling pathways with clinical observations of toxin accumulation. In this way, this narrative review provides a structured link between mechanistic and clinical domains. Experimental data indicate that uremic toxins can interfere with key insulin signaling pathways, whereas clinical studies mainly demonstrate associations rather than causality [12,13]. Within this context, uremic toxins are best considered as amplifiers of pre-existing metabolic disturbances rather than primary drivers of IR. Important limitations remain, including the lack of causal evidence in humans, uncertainties regarding the relevance of doses between experimental and clinical settings, and substantial interindividual variability in microbiome composition and toxin handling [4,14].

## 2. Accumulation and Pathophysiological Impact of Gut-Derived Uremic Toxins

CKD and DKD are increasingly recognized as systemic disorders characterized by bidirectional interactions among renal dysfunction, metabolic disturbances, and gut dysbiosis. In CKD and DKD, this gut dysbiosis is characterized by a shift toward proteolytic microbial metabolism, with expansion of bacterial taxa capable of generating uremic toxin precursors from dietary amino acids, including tryptophan and tyrosine. At the same time, there is a reduction in short-chain fatty acid-producing bacteria, which normally exert anti-inflammatory and metabolic regulatory effects. These compositional and functional changes have been associated with increased production of gut-derived uremic toxins, including IS, PCS, and TMAO. Clinical and translational studies support this link. Microbiome analyses in CKD patients have demonstrated enrichment of urease-, uricase-, and indole-producing bacterial species, alongside depletion of beneficial saccharolytic taxa. These alterations correlate with circulating levels of protein-bound uremic toxins, suggesting that microbial composition directly influences toxin burden. In patients with DKD, similar patterns of dysbiosis have been observed and are associated with metabolic disturbances, including IR and systemic inflammation, although causal relationships remain to be established [15,16,17].

Gut-derived uremic toxins may therefore represent a central interface between these systems. These toxins result from the intestinal microbiome breaking down amino acids and choline from food into indole (from tryptophan), p-cresol (from tyrosine/phenylalanine), and trimethylamine (from choline/L-carnitine), which are then converted into IS, PCS, and TMAO, respectively. The major (protein-bound) uremic toxins are IS and PCS, which are produced from tryptophan and tyrosine/phenylalanine fermentation, respectively. A further important uremic toxin is TMAO, produced by the microbiome via the conversion of trimethylamine (TMA) to TMAO from choline or l-carnitine, followed by hepatic oxidation [14,18]. Importantly, the mechanistic pathways discussed below are largely derived from in vitro and animal models, whereas evidence in human studies is predominantly observational and should be interpreted accordingly.

In health, these metabolites are efficiently cleared by glomerular filtration and tubular secretion. However, as kidney function declines, the clearance of such solutes becomes progressively impaired, leading to their systemic retention and toxic effects. Protein-bound toxins, such as IS and PCS, are particularly problematic because their high affinity for albumin restricts filtration and limits removal even during hemodialysis [19]. In advanced CKD, circulating total IS concentrations may increase from <1 µM in healthy individuals to 50–100 µM, while PCS levels can rise by more than 20-fold compared with controls [20,21,22]. Notably, the plasma concentrations of IS and PCS observed in stage 4–5 CKD overlap with those used in experimental models that demonstrate direct impairment of insulin receptor signaling. Experimental models generally use concentrations higher than those observed in human circulating serum. This creates large gaps in interpreting the meaning of these data for both absolute and relative dose-response relationships, as well as for their significance for translation to humans. To interpret mechanistic data, it is important to distinguish among the three types of exposure (physiological, pathophysiological, and supraphysiological) as they relate to toxic agents. Renal tubular transporters that actively secrete solutes, including organic anion transporters (OAT1 and OAT3), are downregulated in CKD, thereby decreasing renal excretion capacity [12]. TMAO, while less sequestered by protein binding than previously mentioned, is also retained in the system due to declining GFR and has been shown in many clinical cohorts to correlate positively with increased risk for both cardiovascular disease and kidney disease [23].

A shift in gut bacteria populations, dysfunctional gut bacteria, decreased fermentation of sugar-based foods, and increased fermentation of protein-based foods has resulted in a large shift in the production of by-products from gut bacteria, such as indole and p-cresol (both derived from aromatic amino acids) and a reduction in the production of SCFAs from gut bacteria (which are necessary for maintaining the integrity of the intestinal lining and reducing inflammation) [4]. Additionally, uremia causes a change in the proteins that are necessary to keep the intestinal barrier intact (tight junction proteins), which allows for the leakage of gut bacteria and their by-products into the circulation, resulting in increased levels of systemic inflammation [3]. Once retained in the circulation, uremic toxins may adversely affect metabolic and vascular function. IS and PCS have been shown in experimental models to increase reactive oxygen species (ROS) generation via activation of NADPH oxidase and mitochondrial dysfunction, resulting in oxidative damage to renal tubular cells (endothelial cells), insulin-sensitive tissues, and other tissues [12]. Oxidative stress may interfere with insulin receptor substrate phosphorylation, leading to reduced protein kinase B (Akt) activation and decreased glucose uptake in experimental systems, mechanisms that are consistent with insulin resistance but not definitively established as causal in humans [13].

In addition, these toxins are also pro-inflammatory stimuli. PCS and IS activate nuclear factor kappa B (NF-κB), leading to increased expression of certain cytokines (tumor necrosis factor-alpha (TNF-α), interleukin (IL)-1β, IL-6). Consequently, this may contribute to chronic, low-level inflammation [18]. The inflammatory signals produced by these cytokines may alter insulin signaling by inducing suppressor of cytokine signaling 3 (SOCS3) and serine phosphorylation of insulin receptor substrate-1 (IRS-1), both of which inhibit downstream insulin signaling [7]. The inflammatory state is also responsible for renal fibrosis and endothelial dysfunction, both of which are typical hallmarks of DKD [14]. Endothelial cells are extremely susceptible to the toxic effects of these metabolites. Studies have shown that IS can impair endothelial function by inducing oxidative stress, thereby reducing nitric oxide (NO) availability and increasing inflammatory signals [24]. IS and TMAO also inhibit endothelial nitric oxide synthase (eNOS) activity and reduce NO availability, leading to vascular stiffness, impaired microcirculation, and oxidative injury [19]. In addition, endothelial dysfunction caused by these metabolites may accelerate glomerulosclerosis and renal ischemia, and this effect will be further exacerbated by decreased blood flow of glucose and insulin to peripheral tissues [23].

**Figure 1:**
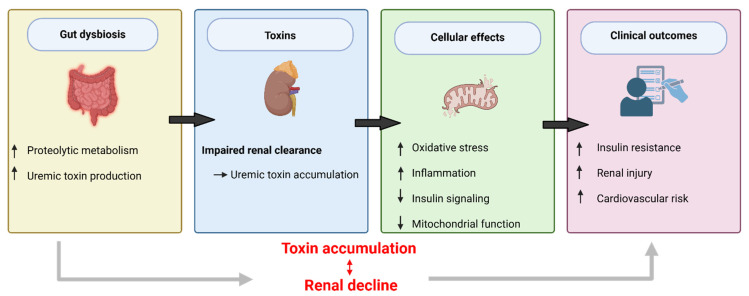
Integrated model of the gut–kidney–metabolic axis in diabetic kidney disease. Gut-based uremic toxins may act as promoters of insulin resistance in diabetic kidney disease (DKD). Gut microbiota disturbances in chronic kidney disease (CKD) and DKD are associated with increased proteolysis, leading to the production of uremic toxin precursors. A systemic accumulation of these toxins characterizes kidney function deterioration. Although very few studies have been carried out in humans, most animal studies have found elevated levels of these toxins capable of triggering oxidative stress, inflammatory processes, and uncontrolled insulin and mitochondrial signaling. These distortions cause cells to become insulin-resistant, lead to kidney tissue damage, and increase the risk of cardiovascular disease. The accumulation of toxins and renal impairment may mutually give rise to each other and contribute to a self-sustaining cycle, which is of great importance for the progression of DKD.

To distinguish between mechanistic and clinical evidence, findings from experimental models are presented alongside observational data from human cohorts. Experimental studies suggest that uremic toxins can impair insulin sensitivity, while clinical studies primarily demonstrate associations between toxin levels and IR. The induction of IR in rodent models by chronic dosing with IS or PCS results in increased homeostatic model assessment of insulin resistance (HOMA-IR) and decreased glucose tolerance. In contrast, antibiotics, prebiotics, or fecal microbiota transplants eliminate toxins and restore insulin sensitivity [25]. In diabetic animals, inhibiting TMAO production reduces inflammation and renal fibrosis, whereas TMAO supplementation has the opposite effect [19]. Human data support these findings. Several studies have shown that patients with CKD/DKD have higher TMAO, IS, and PCS levels in their blood than non-diabetic individuals and that these elevations correlate with both increased HOMA-IR and fasting insulin levels [13]. However, these associations are not entirely consistent across studies, likely reflecting differences in study populations, CKD stages, analytical methods, and adjustment for confounding variables. The relative contribution of individual toxins to metabolic outcomes, therefore, remains difficult to disentangle. In addition, higher TMAO levels are associated with both accelerated progression of eGFR and higher mortality in CKD patients, independent of traditional predictor variables [23]. Higher circulating IS corresponds to higher mortality and vascular disease burden, supporting the toxic accumulation relationship between renal function decline and CKD death [26]. Importantly, most available clinical data are observational, limiting causal inference and raising the possibility of reverse causation, as declining renal function itself leads to toxin accumulation. Though few effective intervention trials exist, some have produced evidence that oral adsorbents such as AST-120 or dietary microbiota-targeted interventions reduce both systemic inflammation and toxin levels in individuals with CKD [14]. Very few randomized controlled trials have measured IR or DKD progression as primary endpoints, but the majority of available clinical evidence remains association-based. In addition, heterogeneity in study design, including differences between experimental models and human cohorts, further complicates interpretation and limits direct translation of findings. Elevated circulating toxin levels reflect impaired renal clearance and do not alone establish causality. Therefore, to determine whether toxins contribute to IR causally will require interventional studies demonstrating that toxin reduction targets lead to improved IR independent of changes in GFR. There is currently insufficient evidence to conclude that uremic toxins causally induce IR in humans. However, the lack of causal evidence should not detract from the conceptual mechanistic unity of what is emerging across experimental models (including those from other species) and human observation cohorts. The cumulative effects of alterations in oxidative, inflammatory, and metabolic signaling across experimental systems and human cohorts are consistent with a biologically plausible integrated model that warrants prospective testing. There is now a growing body of experimental and observational evidence suggesting that a self-perpetuating cycle may exist in DKD, in which declining renal function is associated with increased systemic accumulation of toxic metabolites that are associated with oxidative stress, inflammation, vascular dysfunction, and impaired insulin signaling, potentially contributing to further renal and metabolic injury (see Figure 1) [18]. This framework offers a mechanistically coherent model of DKD pathophysiology. For instance, the plasma levels of IS, PCS, and TMAO can be used as early indicators of DKD severity and metabolic risk, and interventions directed towards decreasing toxic metabolite production, absorption, and/or downstream signaling pathways (including microbiome modification, absorption therapy, and between anti-inflammatory and antioxidant interventions) should be considered as valuable new approaches to help mitigate the interrelated renal/metabolic effects of DKD [4,14]. While these findings support biological plausibility, it is important to emphasize that human data are predominantly observational and do not establish a causal relationship between toxin levels and IR.

## 3. Molecular Pathways Linking Gut-Derived Uremic Toxins to Insulin Resistance

The molecular mechanisms described in this section are primarily derived from in vitro and animal studies, whereas their clinical relevance in humans remains supported mainly by associative evidence. Insulin signaling is initiated by ligand binding to the insulin receptor. The insulin receptor then autophosphorylates and subsequently tyrosine-phosphorylates insulin receptor substrates (IRS-1 and IRS-2). The next step is phosphatidylinositol 3 kinase (PI3K), which, upon activation, leads to the phosphorylation and activation of Akt. Akt then regulates glucose transporter type 4 (GLUT4) translocation, glycogen synthesis, lipid metabolism, and the inhibition of gluconeogenesis. Disruption at any step, particularly inhibitory serine phosphorylation of IRS-1 or impaired Akt activation, results in IR. It has been suggested that gut-derived uremic toxins have been shown in experimental models to disturb this signaling network both directly and indirectly. In the case of DKD, this kind of signaling impairment may represent a contributing mechanism within a broader pathophysiological context. In addition to metabolic abnormalities, IR also potently induces glomerular hyperfiltration, podocyte stress, and profibrotic signaling. Toxin-mediated signaling disruption may represent a contributory mechanism linking metabolic dysregulation to structural kidney injury [27].

Certain proteins bound to uremia can damage insulin signaling pathways through one or more mechanisms. A major scientific discovery was made by Koppe et al. They demonstrated the chronic effects of PCS on CKD mice. Chronic exposure to PCS was associated with increased IR. It reduced skeletal muscle glucose uptake, mediated by activation of extracellular signal-regulated kinases 1 and 2 (ERK1/2) signaling pathways, leading to increased inhibitory serine phosphorylation of IRS-1 and reduced downstream signals activated via the Akt pathway [28]. This research represents one of the first publications to document a mechanism by which a specific gut-derived uremic toxin can impair insulin-mediated metabolic signaling. However, these findings are derived from animal models under controlled conditions and may not fully reflect the complexity of human CKD, including differences in toxin exposure and metabolic context. While these findings provide mechanistic insight, they originate from animal models and may not fully reflect human pathophysiology.

Another major protein-bound toxin is IS, a metabolite of tryptophan metabolism. IS, like other proteins, binds toxins and induces oxidative stress in cells. IS was shown to promote NADPH oxidase and enhance mitochondrial ROS generation in several cell types, including endothelial and skeletal muscle cells [29,30,31]. In addition to oxidative stress pathways, IS exerts key effects by activating the aryl hydrocarbon receptor (AhR), a ligand-activated transcription factor involved in xenobiotic sensing and inflammatory signaling. AhR activation by IS promotes oxidative stress, endothelial dysfunction, and pro-inflammatory gene expression, thereby linking toxin accumulation to vascular and metabolic injury [32,33]. This receptor-mediated mechanism provides a more specific molecular framework for IS signaling beyond generalized oxidative stress. Excessive ROS levels activate stress kinases, such as c-Jun N-terminal kinase (JNK) and p38 mitogen-activated protein kinase (p38 MAPK), which, in turn, phosphorylate IRS-1 at inhibitory serine residues, thereby potentially limiting PI3K/Akt signaling, the major pathway of insulin action. Literature reviews of IR mechanisms in CKD point to oxidative stress-mediated IRS impairment as a key pathway linking uremia with metabolic disorder [12]. Moreover, toxin concentrations used in experimental systems often exceed those observed in human plasma, complicating the interpretation of dose-response relationships and translational relevance.

In addition to oxidative stress, chronic low-grade inflammation contributes to IR. Furthermore, long-term low-grade inflammation caused by toxins entering the bloodstream from the gut is also an established contributor to insulin-resistant states. The actions of IS and PCS activate NF-kB, leading to increased production of the pro-inflammatory cytokines TNF-α and IL-6 [18]. These same cytokines also enhance SOCS, particularly SOCS3 levels, leading to their binding to IRS-1, causing its ubiquitination and subsequent degradation and disconnecting insulin receptor activation from downstream signal transduction cascades. The role of inflammatory cytokine signaling in CKD-associated IR has been extensively reviewed [7], reinforcing the mechanistic plausibility that toxin-induced inflammation may disrupt metabolic pathways. Beyond classical cytokine-mediated inflammation, uremic toxins have been shown to activate innate immune signaling pathways. Toll-like receptor 4 (TLR4), a key damage-associated molecular pattern (DAMP) receptor, is activated by the uremic milieu and contributes to NF-κB–dependent inflammatory responses. Furthermore, recent studies indicate that IS, PCS, and TMAO can activate the NOD-, LRR- and pyrin domain-containing protein 3 (NLRP3) inflammasome, leading to caspase-1 activation and release of interleukin-1β and interleukin-18. This inflammasome-mediated response represents an important link between metabolic stress, innate immunity, and tissue injury in CKD and DKD [34,35,36].

TMAO, like IS and PCS, has been associated with metabolic dysregulation despite its structure being quite different from those of the other two compounds. Experimental studies and observational human data suggest that elevated TMAO levels have been linked to compromised glucose tolerance and IR [23]. Mechanistically, TMAO enhances oxidative stress and activates inflammatory signaling cascades, potentially impairing insulin receptor substrate phosphorylation and reducing Akt signaling efficiency. A systematic review of TMAO in CKD populations further underscores its association with metabolic and cardiovascular risk [37].

Uremic toxin accumulation may also impair renal insulin signaling. Insulin influences the kidneys by regulating sodium handling, promoting the conversion of non-carbohydrate substrates into glucose, and affecting kidney cell survival. When defective insulin signaling is taken into account, it leads to tubules that are unable to transport appropriately (through altered tubular transport) and are more fibrotic (due to greater amounts of fibrotic material). Experimental evidence suggests that uremic milieu and toxin accumulation may interfere with renal insulin signaling pathways, potentially amplifying local oxidative stress and inflammation [12]. Such local IR may exacerbate DKD by promoting mesangial expansion and extracellular matrix deposition.

A second emerging mechanism is mitochondrial dysfunction. IR has been shown to reduce mitochondrial biogenesis and impair electron transport chain function, thereby decreasing ATP production and increasing ROS production [31,38]. Insulin signaling may maintain metabolic flexibility through proper mitochondrial function. Mitochondrial stress may further amplify insulin resistance. Additionally, mitochondrial ROS generation may sustain the ongoing activation of redox-sensitive transcription factors, including NF-kB, thereby creating a continuous loop of inflammation.

Epigenetic modulation represents an additional layer of regulation. Although less extensively characterized, IS exposure has been associated with altered expression of genes regulating oxidative stress and fibrosis via epigenetic mechanisms, including histone modifications and DNA methylation [18]. Such alterations may have long-term effects on metabolic gene expression, potentially stabilizing insulin-resistant phenotypes in CKD and DKD. Emerging evidence suggests that uremic toxins may also exert systemic effects through extracellular vesicles, including microvesicles and exosomes, which carry bioactive molecules such as microRNAs. These vesicles can propagate inflammatory and metabolic signals between tissues, potentially amplifying insulin resistance and vascular dysfunction. Toxin-induced alterations in microRNA expression have been implicated in endothelial dysfunction, fibrosis, and metabolic dysregulation, although their precise role in DKD remains to be fully elucidated [35,36].

Collectively, these molecular pathways converge on a common outcome: impaired IRS-PI3K-Akt signaling, activation of stress kinases (ERK, JNK, p38 MAPK), chronic inflammation, oxidative damage, and mitochondrial dysfunction (Table 1). Importantly, available experimental and translational data suggest a hierarchical organization of these pathways. Oxidative stress appears to function as an important upstream mechanism triggered by toxin accumulation, initiating stress kinase activation and mitochondrial dysfunction. Inflammatory signaling, particularly NF-κB–SOCS3 activation, acts as a secondary amplifier, stabilizing inhibitory IRS-1 phosphorylation. Endothelial dysfunction and vascular rarefaction then propagate systemic metabolic impairment. This hierarchical model provides a framework for prioritizing therapeutic targets within the gut–kidney-metabolic axis [26,28,39]. Overall, these pathways provide biological plausibility, but their causal contribution to IR in humans remains to be established.

The integrated disruptions lead to reduced GLUT4 translocation resulting in decreased glucose uptake in the skeletal muscle and fat, increased hepatic glucose production (gluconeogenesis) to counteract the decrease in blood glucose, and alterations of the insulin response at the kidneys which are also part of this integrated disruption caused by the gut-derived uremic toxins (due to already present hyperglycemia and metabolic stress in DKD patients). Therefore, these processes may contribute to the amplification of systemic IR and kidney injury via an additive effect. Uremic toxin-related IR may represent a second layer of amplification, and partially reversible, toxin-driven processes superimposed on existing metabolic dysfunction. Therefore, further studies using the hyperinsulinemic–euglycemic clamp technique and comprehensive uremic toxin profiling will be required to define the relative contribution of toxin accumulation to whole-body insulin resistance [40]. While these findings support biological plausibility, clinical studies remain observational and do not establish causality. Overall, the literature is characterized by substantial heterogeneity in study design, ranging from in vitro experiments and animal models to observational human studies, each with distinct limitations. This variability complicates direct comparison across studies and limits the strength of translational conclusions.

## 4. Therapeutic Strategies Targeting Gut-Derived Uremic Toxins and Insulin Resistance in DKD

Notwithstanding the aforementioned strong mechanistic rationale, the clinical translation of toxin-targeted therapies remains limited. Most available interventions have demonstrated reductions in circulating uremic toxin levels. However, robust evidence linking these reductions to improvements in IR or hard renal outcomes is currently lacking. Current data are largely derived from small, short-term studies that rely on surrogate endpoints and should therefore be interpreted with caution. In addition, toxin-related therapeutic concepts should be interpreted within the broader contemporary DKD treatment landscape, in which incretin-based therapies and nonsteroidal mineralocorticoid receptor antagonists have expanded the spectrum of potential cardiorenal interventions.

Despite renin–angiotensin system blockade, SGLT2 inhibitors, and GLP-1 receptor agonists, DKD often progresses. The gut–kidney-metabolic axis is gaining increasing attention, and thus therapeutic strategies to reduce gut-derived uremic toxin burden or to attenuate their metabolic effects seem to be the current focus of research. These interventions can generally be divided into (i) changing the gut microbiota composition and metabolic output, (ii) decreasing the intestinal toxin absorption, (iii) increasing toxin clearance, and (iv) focusing on the downstream oxidative, inflammatory, and insulin signaling mechanisms.

Modulation of the gut microbiota represents a rational therapeutic strategy. Both CKD and DKD have been linked to dysbiosis, biochemically characterized by the expansion of proteolytic bacterial species and the reduction of SCFA-producing organisms [14]. Fibers can play a great role in this process through their fermentation by the gut microbiota. Increased saccharolytic fermentation following higher dietary fiber intake results in elevated SCFA production, enhanced epithelial barrier integrity, and reduced systemic inflammation. An in-depth review of the kidney–gut axis reveals that dietary fiber supplementation reduces circulating levels of PCS and IS in patients with CKD [4].

The use of pre- and probiotics as microbiome-directed therapies has been studied. In randomized trials of patients with CKD, synbiotic supplementation has been shown to lower levels of PCS and certain inflammatory markers. Meta-analyses [41,42,43] show that microbiome-focused therapies produce modest reductions in uremic toxin concentrations. However, these studies must be interpreted with caution, as they are generally limited by small sample sizes, short follow-up periods, and a lack of clinically meaningful endpoints, such as insulin sensitivity or DKD progression. One possible way to reduce proteolytic fermentation activity is through microbiome-based therapies that inhibit proteolysis. However, another mechanism likely occurs through stimulation of SCFAs, which exert anti-inflammatory and insulin-sensitizing effects via G protein-coupled receptors (GPR41/GPR43). Microbiome-based therapies also inhibit histone deacetylase activity.

Dietary interventions represent another promising approach. Very-low-protein diets supplemented with ketoanalogues have been shown to reduce uremic toxin production and systemic inflammation in CKD [44]. Plant-based dietary patterns decrease TMAO formation by reducing intake of choline and L-carnitine-rich animal products and altering microbiota composition. A systematic review of TMAO in CKD populations underscores the association between dietary precursors and circulating TMAO levels [37]. Importantly, plant-based diets may simultaneously improve insulin sensitivity, suggesting dual metabolic and renal benefit.

Another way of treating patients with uremia may be through the intestinal absorption of uremic toxins (or their precursors). However, clinical evidence supporting intestinal toxin adsorption strategies remains limited and inconsistent, with most studies relying on surrogate biochemical endpoints rather than hard clinical outcomes. AST-120 is an oral therapeutic charcoal adsorbent that binds indole to the intestinal surfaces to prevent the liver from converting it into IS. Clinical trials have shown that AST-120 has lowered IS levels in people with uremia and may slow the progression of CKD in selected patients [18]. Although large phase III clinical studies [45,46,47] investigating clinical endpoints related to CKD progress or loss of kidney function have yielded inconsistent results, the finding that biomarker concentrations reduce, along with preclinical studies investigating the mechanisms of action of AST-120, suggests that further study of this treatment in metabolically high-risk patients with DKD is warranted. Importantly, reductions in circulating toxin levels have not consistently translated into improvements in hard clinical endpoints, highlighting the gap between mechanistic rationale and clinical efficacy. Discrepancies between reductions in uremic toxins and significant improvements in clinical outcomes may be explained by multiple confounding factors, including incomplete uremic toxin reduction, treatment initiated too late, variability in the microbiome, and the multifactorial nature of IR in CKD. A precise approach will therefore be required to better characterize a population of patients who would benefit from uremic toxin-directed therapies that improve insulin signaling. This subpopulation of patients with a high percentage of circulating, free uremic toxins, preserved beta-cell function, and early CKD may derive a greater biologically relevant benefit from uremic toxin-directed interventions. Future clinical trials evaluating the efficacy of uremic toxin-directed therapies will need to include stratification by uremic toxin levels, analysis of free versus total fractions of uremic toxins, and incorporation of surrogate metabolic end-points to identify responders. It is plausible that toxin-directed therapy must be combined with established renoprotective agents to achieve clinically meaningful benefit. Stratification of patients by baseline toxin burden or metabolic phenotype may enhance the detection of therapeutic signals in future trials.

Direct intervention to oxidative stress and inflammation caused by uremic toxins also constitutes a rational therapeutic strategy. Clinically, supplementation with antioxidants, including N-acetylcysteine, resveratrol, and polyphenols, has been shown to reduce oxidative stress markers in patients with CKD [48]. It is well known that one of the major effects of toxins, the induction of ROS, dysregulates IRS, PI3K, and Akt signaling. Therefore, reducing oxidative stress may partially improve insulin signaling pathways at the molecular level. Furthermore, targeting inflammation with drugs that inhibit NF-κB activation or cytokine signaling can limit SOCS-mediated negative regulation of the insulin receptor, thereby enhancing its response [7].

SGLT2 inhibitors, now a foundational therapy in DKD, may indirectly influence the gut–kidney axis by modulating systemic inflammation and possibly microbial–metabolic signaling [49]. However, their established clinical value in DKD is based primarily on robust cardiorenal outcome trials rather than direct evidence of uremic toxin modulation. In parallel, GLP-1 receptor agonists have emerged as an important therapeutic class in DKD. Current KDIGO guidance recommends a long-acting GLP-1 receptor agonist in adults with type 2 diabetes and CKD who have not achieved glycemic targets despite metformin and an SGLT2 inhibitor, or who cannot use those agents. More recently, the FLOW trial showed that semaglutide reduced the risk of major kidney disease events and death from cardiovascular causes in patients with type 2 diabetes and CKD, supporting a clinically meaningful renoprotective effect beyond glucose lowering. Nonsteroidal mineralocorticoid receptor antagonists, particularly finerenone, represent another important advance. In FIDELIO-DKD and FIGARO-DKD, finerenone reduced kidney and cardiovascular outcomes in patients with CKD and type 2 diabetes, and KDIGO now suggests their use in patients with persistent albuminuria despite optimized renin-angiotensin system blockade. Although these agents are not per se toxin-directed therapies, they are highly relevant to the present framework because they target inflammatory, fibrotic, and metabolic pathways that overlap with the downstream consequences of uremic toxin accumulation [23,50]. By contrast, the evidence supporting the use of DPP-4 inhibitors for DKD modification remains limited. Although these drugs are generally safe in CKD and may modestly reduce albuminuria, meta-analyses have not shown a persuasive benefit on hard renal outcomes or mortality, so their role remains largely one of glycemic management rather than kidney protection. Glucokinase activators are an even earlier-stage concept in this setting. They are of mechanistic interest because of their glucose-lowering potential and have been discussed as possible adjunctive therapies in broader reviews of novel antidiabetic agents, but direct evidence for kidney protection in DKD is currently insufficient. Accordingly, both DPP-4 inhibitors and glucokinase activators should be presented as emerging or supportive perspectives rather than established renoprotective therapies [51].

Finally, emerging therapeutic concepts include fecal microbiota transplantation and precision microbiome engineering. While data in CKD remain limited, early experimental studies suggest that reshaping microbial ecology can significantly alter systemic metabolite profiles, including uremic toxins [25]. Whether such interventions can sustainably improve IR in DKD remains to be determined. Collectively, toxin-directed strategies may complement established DKD therapies (Table 2). Despite promising mechanistic insights, the clinical evidence base remains limited. Most interventional studies are characterized by small sample sizes, short follow-up durations, and reliance on surrogate biochemical markers rather than hard clinical endpoints such as decline in eGFR, onset of end-stage kidney disease, or validated measures of insulin sensitivity.

Furthermore, heterogeneity in study populations and interventions limits comparability across trials. As a result, the clinical relevance of reducing uremic toxin levels remains uncertain. Within this broader therapeutic landscape, the strongest current outcome data support SGLT2 inhibitors, GLP-1 receptor agonists, and nonsteroidal mineralocorticoid receptor antagonists, whereas toxin-directed interventions, DPP-4 inhibitors, and glucokinase activators remain less well validated for hard renal or metabolic endpoints. Overall, while targeting gut-derived uremic toxins represents a biologically plausible therapeutic strategy, current interventions lack sufficient clinical validation. In particular, their impact on IR and the progression of DKD remains uncertain and requires confirmation in adequately powered, long-term randomized trials with appropriate metabolic and renal endpoints.

## 5. Biomarkers and Clinical Implications of Gut-Derived Uremic Toxins in Diabetic Kidney Disease

Identifying accurate biomarkers that detect metabolic dysfunction and kidney injury is an important unmet need in the field of DKD. Traditional clinical measures such as estimated glomerular filtration rate (eGFR) and albuminuria provide some insight into prognosis. However, they do not capture all the metabolic and inflammatory aspects of disease progression. Recently, the presence of gut-related uremic toxins such as IS, PCS, or TMAO has provided insight into the mechanisms at work in DKD, and these substances are also being studied as possible circulating biomarkers that can demonstrate directional activity along the gut–kidney–metabolic axis.

In a cross-sectional study of 149 patients with stage 3–4 CKD, Rossi et al. reported that both free and total IS were independently associated with IL-6, TNF-α, and interferon-gamma (IFN-γ), whereas PCS was associated with IL-6 and pulse wave velocity. Free fractions of both toxins were also associated with glutathione peroxidase activity, supporting links with inflammation, oxidative stress, and vascular stiffness [52]. In addition, circulating PCS and IS increase progressively across CKD stages [53], while Wu et al. reported that higher baseline concentrations were associated with faster CKD progression [22]. Barreto et al. showed that higher serum IS was associated with vascular disease and all-cause mortality in CKD patients. These observations support the view that toxin burden may reflect not only reduced renal clearance but also a higher-risk renal and cardiovascular phenotype [26].

Since TMAO is linked to significant risks, such as cardiovascular disease and metabolic problems, there has been growing interest in its use as a biomarker. In an observational study, baseline TMAO concentrations in patients who eventually developed CKD were much higher than those before progression to CKD. Furthermore, TMAO levels were significantly greater in patients with progressively worsening renal function over time [23]. Meta-analyses also support the association between elevated TMAO levels and increased all-cause mortality in CKD populations [37]. Moreover, since cardiovascular disease is the most common cause of mortality for patients with DKD, TMAO is likely to be a clinically relevant integrative biomarker linking metabolic, renal, and vascular risk.

Dynamic evaluation of toxin levels is an effective way to assess the prognosis of individuals with toxin-related issues. This dynamicity can be helpful for IS or PCS serum levels and their responses to dietary or drug-based changes. Several studies examining the treatment of the microbiota using therapeutic methods have reported significant reductions in toxin levels, which, in turn, have led to improvements in inflammatory markers [14]. Although large-scale validation is lacking, these findings support the concept of toxin profiling as a monitoring tool in clinical practice.

At the mechanistic level, direct clinical evidence linking specific protein-bound uremic toxins to IR indices remains limited. Available observational studies and recent clinical cohort data suggest that a higher circulating burden of phenolic uremic toxins is associated with hyperinsulinemia-related phenotypes in CKD and DKD, but toxin-specific associations with HOMA-IR or fasting insulin have not yet been consistently established across well-characterized human cohorts [13]. Accordingly, the current literature shows an association between uremic toxin accumulation and metabolic dysfunction, rather than a direct, reproducible relationship between individual toxins and IR metrics. Recent data further highlight the complexity of interactions between toxins and metabolism. A clinical study in patients with DKD reported that circulating phenyl sulfate levels were negatively associated with hyperglycemia but positively associated with hyperinsulinemia, independent of kidney function. These findings suggest that specific uremic toxins may reflect distinct metabolic phenotypes, potentially related to compensatory insulin secretion rather than overt glycemic control, and underscore the heterogeneity of toxin–metabolic relationships in CKD and DKD [11]. Such correlations suggest that toxin levels may reveal more about metabolic dysfunction than glycemic indices alone. Hence, combining toxin levels with risk, stratification models may lead to better pinpointing of diabetic kidney disease patients who are at greatest risk of metabolic and renal worsening.

Along with individual toxins, composite biomarker panels combining inflammatory cytokines, oxidative stress markers, and microbial metabolites can illustrate a more complete picture of disease activity. Opinions in the literature regarding the kidney–gut axis suggest that combining metabolomic profiling with conventional renal parameters could identify high-risk phenotypes at an early stage [4]. Advances in mass spectrometry-based metabolomics now allow precise quantification of protein-bound solutes, facilitating clinical translation.

Nevertheless, there are still several obstacles to overcome for the regular use of such a measurement. Firstly, it is of utmost importance to establish standardized protocols for determining protein-bound toxins, as variability in sample preparation and analysis methods leads to inconsistent results. In addition, several confounding factors affect circulating levels, including diet, antibiotic treatment, and the composition of the patient’s microbiome. Further prospective study designs may help identify clinically relevant toxin levels and verify the relationship between treatment based on toxin concentration and better clinical outcomes [18,21,54].

Uremic toxins from the gut are likely to represent a link between basic science and clinical practice, as they can be measured in circulation, exert causal effects on IR pathways, and are associated with outcomes in the kidney and cardiovascular system. They are thus potential clinical biomarkers for precision medicine. Profiling for uremic toxins in clinical trials could improve our ability to identify individuals and patient subgroups most likely to respond to microbiota-targeted therapies or anti-inflammatory treatments [21,22,26,55]. However, variability in analytical methods, dietary influences, and microbiome composition limits standardization and may affect clinical applicability.

## 6. Translational and Clinical Implications

Despite extensive mechanistic evidence, the translation of toxin-mediated pathways into clinical decision-making remains limited. At present, uremic toxins such as IS, PCS, and TMAO should be viewed primarily as risk markers rather than validated therapeutic targets. Their measurement may contribute to patient stratification in both clinical research and potentially future practice.

From a clinical perspective, several patient subgroups may be hypothesized to derive greater benefit from toxin-directed approaches: (i) individuals with early-stage CKD or DKD, where metabolic disturbances may still be modifiable; (ii) patients with disproportionately high circulating toxin levels relative to eGFR; and (iii) patients with prominent IR not fully explained by classical metabolic factors. In these populations, toxin profiling could help identify a phenotype characterized by combined metabolic and uremic burden.

Nevertheless, important limitations currently preclude routine clinical implementation. First, standardized thresholds for toxin levels have not been established. Second, inter-individual variability driven by diet, microbiome composition, and analytical methodology limits reproducibility. Third, and most importantly, there is no conclusive evidence that toxin-guided interventions improve hard clinical outcomes, including insulin sensitivity, renal progression, or cardiovascular events.

Consequently, the primary clinical utility of uremic toxins at present lies in risk stratification and hypothesis generation, rather than direct therapeutic guidance. Future interventional trials should incorporate toxin-based stratification, alongside metabolic phenotyping (e.g., HOMA-IR or clamp-derived indices), to determine whether specific patient subgroups benefit from targeted interventions. Until such data are available, the impact of toxin-directed strategies on clinical decision-making remains uncertain.

## 7. Future Perspectives and Emerging Research Directions in the Gut–Kidney–Metabolic Axis

Future research should focus on connecting mechanistic insights and clinical relevance by integrating molecular, metabolic, and microbiome-derived data in well-characterized human populations. A key unresolved issue is whether uremic toxins act as causal mediators or as amplifiers of insulin resistance, given that current human evidence remains largely associative and experimental models may not reflect physiological exposure levels [12,13]. Addressing dose–response relationships and interindividual variability, including differences in microbiome composition and toxin handling, will be essential for improving translational relevance [4,14]. In addition, emerging data indicate that not all uremic solutes exert uniform metabolic effects. This suggests that a broader, systems-level perspective beyond canonical toxins is needed [11]. Overall, longitudinal and interventional human studies are required to clarify the extent to which toxin-mediated pathways contribute to insulin resistance in DKD.

The gut–kidney axis and gut-derived uremic toxins that contribute to the development of IR and DKD are still not well understood, and many important knowledge gaps remain that must be bridged to develop precision medicine solutions that change the trajectory of these diseases based on mechanistic findings.

The use of integrated multi-omics approaches will also drive the future trajectory of investigations in this area. High-resolution investigations of the microbiome and related metabolites in patients with CKD are now possible thanks to the development of cutting-edge technologies, including metabolomics, metagenomics, and transcriptomics. Recent advances in untargeted metabolomics have identified distinct toxin signatures that associate with varying degrees of severity and progression of CKD [4].

Although the current probiotic and prebiotic interventions demonstrate only slight decreases in toxin levels [41,42,43], new methods are being developed to target the enzymes responsible for toxin production. For example, animal models have shown that inhibitors of microbial TMA lyases can decrease TMAO production [49]. Perhaps subsequent studies might focus on enzyme-specific inhibitors or phage therapies targeting the bacteria that produce indole and p-cresol. However, long-term safety, ecological stability, and unintended alterations to the microbiome require rigorous evaluation.

Mitochondrial-targeted therapies represent an emerging area of interest. Previously, the mechanisms by which toxins, such as IS, can cause mitochondrial dysfunction and ROS production [31,38]. Mitochondria, targeted antioxidants such as MitoQ, and other redox-modulating agents can neutralize the toxin, preventing oxidative damage right at the source. These compounds have not yet been broadly tested in patients with DKD. However, preclinical studies indicate benefits in improving metabolic flexibility and insulin sensitivity [56,57].

The relationship between SGLT2 inhibitors and the gut–kidney axis warrants further exploration. While SGLT2 inhibitors largely protect the kidneys through hemodynamic and metabolic pathways, new evidence suggests that they may also influence gut microbiota composition and systemic inflammation [58,59]. It is unclear if some of their insulin-sensitizing or anti-inflammatory benefits are due to indirect effects on uremic toxin generation, but this is a promising topic for future translational study.

Artificial intelligence and machine learning-based modeling may also enhance the prediction of toxin-related risk. By integrating microbiome composition, metabolomic signatures, inflammatory biomarkers, and clinical parameters, predictive algorithms could identify individuals most likely to develop rapid DKD progression. Re-evaluations of the kidney–gut axis suggest that combining multidimensional biomarkers may outperform standard single-marker techniques [14]. Crucially, surrogate signs must not be the only focus of future clinical studies. Few robust randomized controlled trials are examining difficult outcomes, including the development of DKD, IR markers, cardiovascular events, or mortality, despite several studies demonstrating reductions in circulating IS or TMAO. Toxin profiling, metabolic endpoints (e.g., clamp studies, HOMA-IR), and longitudinal assessment of renal function should all be included in future interventional trials.

Microbial ecology and variations in toxin production are influenced by host genetics, nutrition, drug exposure (including metformin and antibiotics), and geographic location. Customized microbiome-based therapies may, therefore, be necessary. Over time, cohort studies tracking microbiome changes across different stages of DKD would be very helpful for understanding the temporal patterns of toxin production [60,61,62,63,64].

Finally, the mechanistic investigation of epigenetic and transcriptional reprogramming caused by prolonged toxin exposure remains unexplored. Chronic activation of inflammatory and oxidative pathways may lead to permanent changes in gene expression, resulting in metabolic memory in DKD [18]. Understanding these epigenetic markers may enable specific correction of toxin-induced metabolic dysfunction.

In summary, subsequent studies on the gut–kidney metabolic axis should integrate multi-omics profiling, precision modulation of the microbiome, mitochondrial-targeted therapies, and pivotal clinical trial design. Gut-derived toxins are increasingly viewed as active components of the metabolic–renal axis. A mechanistic view fostered by novel therapeutic approaches may, in the end, help attenuate the proposed feed-forward interaction between dysbiosis, toxin accumulation, metabolic impairment, and renal decline. Despite accumulating evidence, several controversies remain. First, whether uremic toxins are true upstream drivers of IR or downstream amplifiers of metabolic dysfunction remains debated. Second, the relative contribution of protein-bound toxins compared with other uremic retention solutes is unclear. Third, the generalizability of mechanistic results is complicated by inter-individual diversity in microbiota composition. Lastly, it remains unclear how much toxin-mediated IR can be reversed with early intervention. Setting treatment priorities within the gut–kidney–metabolic axis will depend on answering these concerns. Designing mechanistic human studies that include longitudinal kidney endpoints, consistent toxin quantification, and gold-standard insulin sensitivity tests will be a crucial next step. Instead of demonstrating a clear translational influence, the discipline risks being limited to association inference in the absence of such investigations.

## 8. Limitations of Current Evidence and Remaining Knowledge Gaps

There are major gaps in our knowledge despite the increasing comprehension of the gut, kidney, metabolic axis, and gut-derived uremic toxins in IR and DKD. While the associative evidence is indeed strong and the molecular plausibility is very solid, the cause-and-effect relationship, clinical application, and therapeutic translation have yet to be fully proven.

The prevalence of observational data is a major drawback. Circulating levels of TMAO, PCS, or IS have been linked to worse renal or cardiovascular outcomes in several cohort studies [23,37]. Nevertheless, it is impossible to determine from observational data whether these metabolites indicate systemic inflammation and deteriorating renal clearance, or the actual causes of IR and the progression of DKD. Reverse causality remains possible, particularly given that reduced glomerular filtration inherently increases circulating toxin concentrations.

Although experimental models provide stronger evidence for causality, translation from animal studies to human disease is challenging. Rodent models have demonstrated that PCS induces skeletal muscle IR via ERK1/2 activation [28] and that IS promotes oxidative stress and mitochondrial dysfunction [31,38]. However, toxin concentrations used in experimental systems may exceed physiological levels observed in human CKD, raising questions about the relevance of the dose-response relationship. Additionally, murine microbiome composition differs substantially from that of humans, limiting direct extrapolation of microbiota-targeted interventions.

Another significant limitation is the heterogeneity of methodologies for measuring toxins. Protein-bound solutes, such as IS and PCS, must be measured using specialized techniques, such as high-performance liquid chromatography or mass spectrometry. Variable findings may arise from variations in sample preparation (total vs. free fraction), storage conditions, and analytical platforms. Reviews have emphasized the need to standardize uremic toxin assays before widespread clinical adoption [18]. Without harmonized measurement protocols, defining universal threshold values for risk stratification remains difficult.

Clinical trials targeting toxin reduction have also produced mixed results. Oral adsorbent therapy with AST-120 reduces circulating IS levels, but large trials have not consistently demonstrated significant slowing of CKD progression in unselected populations [18]. It remains unclear whether specific subgroups, such as patients with high baseline toxin burden or pronounced IR, may derive greater benefit. Similarly, probiotic and synbiotic treatments have demonstrated slight decreases in inflammatory and PCS markers [41], although long-term metabolic and renal outcomes are rarely assessed.

The interaction among medicine, nutrition, and the microbiome adds another layer of complexity. Microbial metabolism and toxin production are influenced by dietary protein intake, fiber intake, red meat intake (a significant source of TMAO precursors), antibiotic exposure, and the use of drugs such as metformin or SGLT2 inhibitors. Plant-based diets, for instance, are associated with lower TMAO concentrations [37], but individual adherence varies widely. It is consequently difficult to separate the effects of nutrition from inherent changes in the microbiome in DKD populations.

Furthermore, other factors contribute to IR in CKD. Adipokine signaling, lipotoxicity, advanced glycation end products (AGEs), and hormonal changes are among the parallel processes through which uremic toxins interact. The cumulative effects of oxidative stress, inflammation, metabolic acidosis, and decreased insulin clearance are highlighted in reviews of IR in CKD [7,12]. Therefore, carefully regulated mechanistic investigations are necessary to isolate the independent impact of gut-derived toxins.

The issue of temporal dynamics is another open subject. The point at which toxin-mediated IR in DKD becomes clinically severe is yet unknown. To determine whether higher toxin levels precede IR or result from advanced renal impairment, longitudinal studies including serial toxin measurements, microbiome profiling, and metabolic evaluation are required.

Finally, most research to date has focused on a limited subset of well-characterized toxins (IS, PCS, and TMAO) while hundreds of additional microbial metabolites may contribute to metabolic dysfunction. Numerous previously overlooked solutes in CKD plasma have been found by thorough metabolomic analysis [4]. Identifying these physiologically active, therapeutically useful compounds will be the focus of future research. Taken together, these limitations indicate that current evidence remains insufficient to establish a causal role for uremic toxins in insulin resistance in humans.

## 9. Conclusions and Integrative Perspective

The convergence of CKD, IR, and DKD reflects a complex network of metabolic, inflammatory, and microbiome-driven interactions that extend beyond traditional paradigms of hyperglycemia-induced renal injury. Accumulating evidence suggests that gut-derived uremic toxins may represent one component of a broader metabolic–renal network, acting as biologically active metabolites that are associated with alterations in metabolic and renal signaling pathways. Clinical data consistently link elevated circulating IS, PCS, and TMAO concentrations with IR indices, a faster decline in eGFR, and a higher mortality risk [13,23]. Importantly, gut-derived toxins may contribute to a mechanistic framework that could partially explain the disproportionately high burden of metabolic syndrome features observed in CKD populations, independent of classical glycemic control.

The kidney is closely integrated with intestinal microbial metabolism and systemic metabolic signaling. In DKD, toxins can alter insulin signaling pathways, accelerating cardiovascular and renal complications when ROS, inflammation, and hyperglycemia are already elevated in the tissue. Among the choices for future studies, longitudinal multi-omics-based studies, including metabolomic profiling, insulin sensitivity testing, inflammatory markers, and microbiome sequencing, are urged to take priority. Before regular clinical use, toxin measurement should be standardized, dose-response relationships should be made clear, and clinically relevant thresholds should be established. Lastly, intestinal dysbiosis, IR, and DKD development are molecularly linked by gut-derived uremic toxins. Through oxidative, inflammatory, and stress kinase pathways, these metabolites may amplify metabolic and renal dysfunction. Targeting this axis represents a potential therapeutic avenue that requires validation in adequately powered clinical studies and moves toward a more integrated understanding of metabolic–renal disease. We suggest that DKD may be viewed, at least in part, as a metabolic disease influenced by the microbiota, with toxin-mediated IR potentially acting as a hypothesized amplifying factor within this network. Importantly, the concepts presented in this review should be considered hypothesis-generating. While experimental studies provide mechanistic plausibility and observational human data demonstrate consistent associations, causal relationships between uremic toxins, IR, and DKD progression remain unproven. The heterogeneity of clinical studies, variability in toxin measurement, and lack of interventional data with hard clinical endpoints currently limit translation into clinical practice. Future research should therefore prioritize well-designed longitudinal and interventional studies to determine whether toxin-targeted strategies confer meaningful metabolic or renal benefit.

## Figures and Tables

**Table 1:** Molecular pathways linking major gut-derived uremic toxins to insulin resistance and diabetic kidney disease progression.

Toxin	Microbial Origin	Accumulation Mechanism in CKD	Molecular Targets	Insulin Signaling Effect	Renal/Vascular Effects	Refs.
IS	Tryptophan → indole	Protein-bound toxin; limited filtration + OAT1/OAT3-dependent secretion; accumulation due to reduced tubular secretion and albumin binding	AhR, NADPH oxidase, NF-κB, NLRP3 inflammasome	ROS-mediated IRS inhibition	Fibrosis, oxidative stress	[28,29,30,31,37,38]
PCS	Tyrosine/phenylalanine → p-cresol	Protein-bound toxin; strong albumin binding; reduced tubular secretion; poor dialysis clearance	ERK1/2, TLR4, NLRP3 inflammasome	IRS-1 serine phosphorylation	Endothelial dysfunction	[28]
TMAO	Choline/L-carnitine → TMA	Water-soluble toxin; freely filtered; accumulation primarily due to reduced GFR	TLR4, NLRP3 inflammasome	Impaired Akt signaling (indirect)	Atherosclerosis, CKD progression	[23,37]

Abbreviations: CKD, chronic kidney disease; IS, indoxyl sulfate; OAT, organic anion transporter; NADPH, nicotinamide adenine dinucleotide phosphate; NF-κB, nuclear factor kappa B; ROS, reactive oxygen species; IRS, insulin receptor substrate; PCS, para-cresyl sulfate; ERK1/2, extracellular signal-regulated kinases 1 and 2; TMAO, trimethylamine-N-oxide; TMA, trimethylamine; Akt, protein kinase B; TLR4: Toll-like receptor 4; NLRP3: NOD-, LRR-en pyrin-domeinhoudend proteïne 3.

**Table 2:** Therapeutic strategies targeting gut-derived uremic toxins in DKD.

Strategy	Mechanism	Evidence Type	Clinical Relevance	Limitations
Dietary interventions	Reduce toxin precursors	Observational + small trials	Supportive	Adherence, heterogeneity
Microbiome modulation (pre/pro/synbiotics)	Alters gut metabolism, reduces toxin generation	Small RCTs, meta-analyses	Modest toxin reduction	No hard evidence
AST-120	Intestinal toxin adsorption	Large RCTs (negative/inconsistent)	Low	No consistent renal benefit
Established DKD therapies (SGLT2i, GLP-1 RA, MRA)	Metabolic, anti-inflammatory, and hemodynamic	Large RCTs	High	Indirect effect on toxins

Abbreviations: RCTs, randomized controlled trials; SGLT2, sodium–glucose cotransporter 2; DKD: Diabetic Kidney Disease; GLP-1 RA: Glucagon-Like Peptide-1 Receptor Agonist; MRA: Mineralocorticoid Receptor Antagonist.

## Data Availability

No new data were created or analyzed in this study. Data sharing is not applicable to this article.

## Abbreviations

The following abbreviations are used in this manuscript:

AGEs	Advanced glycation end products
Akt	Protein Kinase B
AST-120	Oral charcoal adsorbent (Kremezin)
CKD	Chronic kidney disease
DKD	Diabetic kidney disease
eGFR	Estimated glomerular filtration rate
eNOS	Endothelial nitric oxide synthase
ERK	Extracellular signal-regulated kinase
ESRD	End-stage renal disease
FMT	Fecal microbiota transplantation
GFR	Glomerular filtration rate
GLP-1	Glucagon-like peptide-1
GLUT4	Glucose transporter type 4
GPR	G-protein-coupled receptor
HOMA-IR	Homeostatic model assessment of insulin resistance
IL	Interleukin
IR	Insulin receptor
IRS	Insulin receptor substrate
IS	Indoxyl sulfate
JNK	c-Jun N-terminal kinase
MAPK	Mitogen-activated protein kinase
NADPH	Nicotinamide adenine dinucleotide phosphate
NF-κB	Nuclear factor kappa B
NO	Nitric oxide
OAT	Organic anion transporter
PCS	p-cresyl sulfate
PI3K	Phosphatidylinositol-3-kinase
ROS	Reactive oxygen species
SCFA	Short-chain fatty acid
SGLT2	Sodium–glucose cotransporter 2
SOCS	Suppressor of cytokine signaling
TMA	Trimethylamine
TMAO	Trimethylamine-N-oxide
TNF-α	Tumor necrosis factor alpha
